# Treatment of Microsporidium *Nosema bombycis* Spores with the New Antiseptic M250 Helps to Avoid Bacterial and Fungal Contamination of Infected Cultures without Affecting Parasite Polar Tube Extrusion

**DOI:** 10.3390/microorganisms12010154

**Published:** 2024-01-12

**Authors:** Igor V. Senderskiy, Viacheslav V. Dolgikh, Diloram A. Ismatullaeva, Bakhtiyar A. Mirzakhodjaev, Anastasiia P. Nikitina, Danil L. Pankratov

**Affiliations:** 1 All-Russian Institute of Plant Protection, Podbelsky Chausse 3, 196608 Saint-Petersburg, Russia; senderskiy@mail.ru; 2 Scientific Research Institute of Sericulture, Ipakchi Str. 1, Tashkent 100069, Uzbekistan; ismatullayeva1972@mail.ru (D.A.I.); uzniish@mail.ru (B.A.M.); 3 Department of Microbiology and Virology, Pavlov First Saint-Petersburg State Medical University, L’vaTolstogo Str. 6-8, 197022 Saint-Petersburg, Russia; anastasiya.nika998@gmail.com (A.P.N.); pdl-19102000d@mail.ru (D.L.P.)

**Keywords:** microsporidia, *Nosema bombycids*, spores, cell culture, M250

## Abstract

Microsporidia are a group of widespread eukaryotic spore-forming intracellular parasites of great economic and scientific importance. Since microsporidia cannot be cultured outside of a host cell, the search for new antimicrosporidian drugs requires an effective antiseptic to sterilize microsporidian spores to infect cell lines. Here, we show that a new polyhexamethylene guanidine derivative M250, which is active against fungi and bacteria at a concentration of 0.5–1 mg/L, is more than 1000 times less effective against spores of the microsporidium *Nosema bombycis*, a highly virulent pathogen of the silkworm *Bombyx mori* (LC_50_ is 0.173%). Treatment of *N. bombycis* spores that were isolated non-sterilely from silkworm caterpillars with 0.1% M250 solution does not reduce the rate of spore polar tube extrusion. However, it completely prevents contamination of the Sf-900 III cell culture medium by microorganisms in the presence of antibiotics. The addition of untreated spores to the medium results in contamination, whether antibiotics are present or not. Since 0.1% M250 does not affect spore discharging, this compound may be promising for preventing bacterial and fungal contamination of microsporidia-infected cell cultures.

## 1. Introduction

Microsporidia (Opisthosporidia: microsporidia) are a large group of fungi-related spore-forming obligate intracellular parasites with the most unusual cell biology among eukaryotes [1,2,3]. The majority of microsporidian species develop in direct contact with the host cytoplasm, and their intracellular stages have acquired the ability to import host-derived ATP through plastidic-bacterial ADP/ATP transporters [4,5,6]. Microsporidia secrete a number of enzymes and regulatory proteins into infected cells to manipulate host metabolic and regulatory pathways [7,8,9,10]. In adapting to their intracellular lifestyle, microsporidia have lost some typical eukaryotic cellular components, such as canonical mitochondria with tricarboxylic acid cycles and respiratory chains [11,12,13,14] and coated vesicles in the Golgi apparatus [15]. Furthermore, these microorganisms have highly reduced genomes [16,17,18], and their ribosomes differ from the common eukaryotic type but resemble prokaryotic ones [19]. This comprehensive study of this group of microorganisms is not only due to the peculiarities of their cell biology but also for economic and medical reasons.

These parasites are widely distributed in the environment, with zoonotic, foodborne, water-borne and transovarial ways of transmission [20]. They can infect an extensive range of animal hosts, and several genera cause infections in humans [21,22,23]. A large number of microsporidian species have an insect as a type of host. There is considerable diversity in the exploitation of their hosts observed, ranging from cryptic, benign infections to massive epizootics with drastic population declines. In human economic activity, entomopathogenic microsporidia are considered to be either as perspective biological control agents of pests or as dangerous pathogens of beneficial insects [24], such as *Bombyx mori* and *Apis mellifera.* The pathogens of these insects have been implicated in causing significant losses to the silk industry and beekeeping.

The first recorded species of microsporidia was *Nosema bombycis* Nägeli, 1857 from the silkworm *B. mori* [25]. Louis Pasteur discovered that this parasite was the etiological agent of the silkworm disease known as pébrine [26]. In the mid-19th century, pébrine almost completely destroyed silk production in France and Italy and remains a threat to the silk industry worldwide. Pasteur’s main strategies for preventing pébrine disease were based on sanitation and disease-free breeding, as *N. bombycis* is transmitted either horizontally or vertically [27]. However, the disinfection of silkworm nurseries and careful microscopic control of silkworm egg producers is not intended to eradicate the parasite completely, but to minimize the spread of infection to a level that is no longer economically significant.

More recently, some chemical drugs have been developed for antimicrosporidian therapy. For example, albendazole has been shown to be effective against several human pathogens, and fumagillin is applied to protect honeybees from *Nosema apis* infection [28]. A carbendazim-based formulation is also used to prevent *N. bombycis* infection in silkworm seed production [29]. An informative paper has recently been published on the treatment of honeybee nosemosis with antibiotics, small organic molecules, extracts and natural compounds [30]. An alternative approach is to create genetically resistant silkworm strains (breeds) using RNA interference, single-chain antibodies or CRISPR/CAS9 technologies [31,32,33].

Since microsporidia cannot be cultivated outside of the host cell and working with a whole host organism is not always feasible, the search for new antimicrosporidian drugs can be performed in artificially infected cell lines. For example, the IPL-LD 65Y cell line infected with microsporidia *N. ceranae*, obtained from the gypsy moth *Lymantria dispar*, has been used to study the effects of chemical compounds such as quinine, surfactin and several nitroimidazoles on the intracellular development of the parasite [34]. Model systems in which microsporidia infect cell lines genetically close to their type hosts are also used to study the molecular biology of these parasites, as well as in research for genetic resistance to them [33]. Infection of cell lines kept under sterile conditions is carried out by microsporidian spores obtained from naturally infected animals whose isolation conditions are usually far from aseptic. Therefore, they must be thoroughly cleaned and antiseptically treated to prevent further viral, bacterial or fungal contamination of the cell culture.

In this study, we demonstrated the efficacy of the novel antiseptic compound M250 (poly-N1-hydrazino(imino)-methyl-1,6-hexanediamine) as a means of sterilizing microsporidian *N. bombycis* spores prior to infection of the *Spodoptera frugiperda* cell line Sf9 in comparison to the standard antiseptic chlorhexidine. We also tested the ability of the antibiotic mixture to prevent bacterial and fungal contamination either with or without antiseptic treatment.

## 2. Materials and Methods

### 2.1. Test Substances

**Figure 1:**
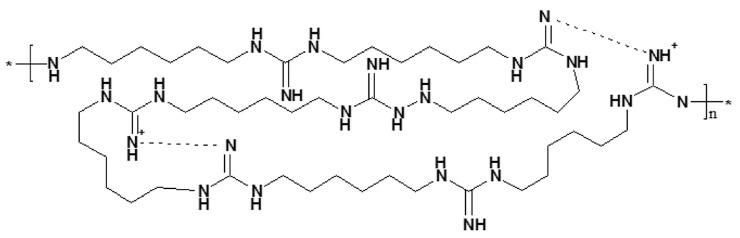
Chemical structure of Mul-1867, the active antimicrobial ingredient in M250. The asterisk denotes the site of attachment to an adjacent repeating unit of the polymer chain.

The experimental compound M250 (Figure 1), obtained from the Human Microbiology Institute (New York, NY, USA), is a new derivative of polyhexamethylene guanidines with high antifungal and antibacterial activities. The compound consists of Mul-1867 (poly-N1-hydrazino(imino)-methyl-1,6-hexanediamine) 2.5% water solution [35]. A 70% stock solution of M250 in sterile distilled water was stored at 4 °C. Working dilutions were prepared ex tempore.

The antibiotic–antimycotic composition contained 1 mg/mL tetracycline hydrochloride (ITW Reagents, Monza, Italy), 0.25 mg/mL chloramphenicol (Sigma-Aldrich, St. Louis, MO, USA), 5 mg/mL gentamicin (Sigma-Aldrich, St. Louis, MO, USA), 10 mg/mL kanamycin sulfate (Gibco, Thermo Fischer Scientific, Waltham, MA, USA) and 25 µg/mL amphotericin B (Gibco, Thermo Fischer Scientific, Waltham, MA, USA). It was used in 100-fold dilutions.

The reference antiseptic chlorhexidine (Rosbio, Saint Petersburg, Russia) was used as 2% and 0.05% water solutions.

### 2.2. Isolation and Treatment of N. bombycis Spores

*N. bombycis* spores were obtained from the Uzbek Research Institute of Sericulture in Tashkent, Uzbekistan. Spores were isolated from artificially infected 5th instar *B. mori* caterpillars under non-sterile conditions. To avoid over-contamination with intestinal microorganisms, only the fat bodies and silk glands of the caterpillars were used. These organs were homogenized in distilled water and then centrifuged at 600× *g* for 5 min. Spore pellets were washed three times in water and additionally purified by centrifugation in the same mode at a density gradient of 50% Percoll (Merck, Darmstadt, Germany), prepared with distilled water at 13,000× *g* for 15 min. A pellet of mature spores was stored in distilled water at 4 °C.

For the antiseptic treatment of spores, approximately 2 × 10^7^ *N. bombycis* spores in 50 µL of aqueous suspension were incubated in the presence of 0.1–1% M250 for 5, 30 or 60 min at room temperature. The same number of spores were incubated in 50 µL of 0.05% or 2% aqueous solution of chlorhexidine for 30 min at room temperature. After incubation, the spores were pelleted at 300× *g* for 5 min and washed three times with 1 mL of sterile distilled water.

### 2.3. Activation and Counting of N. bombycis Spores

Spores were treated, washed and finally pelleted at 300× *g* for 5 min and resuspended in 50 µL of 10 mM KOH. They were activated under alkaline conditions for 2–15 min, after which 5 µL of the suspension was mixed with 35 µL of phosphate-buffered saline (PBS; 138 mM NaCl, 3 mM KCl, 1.5 mM KH_2_PO_4_, 8 mM Na_2_HPO_4_, pH 6.8) on a microscope slide where the spore discharge occurred [36]. It should be noted that spores kept in 10 mM KOH during the day retained the ability to extrude polar tubes.

Polar tube extrusions were observed at 1000× magnification in the phase-contrast optics of an AxioImager M1 microscope (Carl Zeiss, Oberkochen, Germany). Dark discharged and light undischarged spores were counted on images obtained with an Axiocam 712 mono camera of the same manufacturer using the Zen 3.2 blue edition software. Each image contained several dozen *N. bombycis* spores.

### 2.4. Contamination Assay

To test the efficacy of antiseptics, we used 3 treatments of *N. bombycis* spores: 0.1% M250, 0.05% and 2% chlorhexidine, and untreated (control) spores. All 4 variants were also repeated with the addition of 100× antibiotic–antimycotic composition containing tetracycline, chloramphenicol, gentamicin, kanamycin and amphotericin B. Each of the variants analyzed was performed three times in 6 different wells of a 48-well plate.

*N. bombycis* spores were treated with an appropriate antiseptic for 30 min, washed 3 times with sterile water and resuspended in 20 µL of 10 mM KOH. After 15 min, 20 µL of 2 × 10^6^ spore suspension was added to each well containing 200 μL of SF900 III culture medium (Thermo Fisher Scientific, Waltham, MA, USA), where polar tube extrusions occurred. The assay was carried out at 27 °C. Contaminating growth of microorganisms was assessed by visual analysis of turbidity and microscopy up to 7 days after the addition of spores to the medium.

### 2.5. Statistical Analysis

All experiments were performed in triplicate. The results (percentage of discharged spores) were provided as mean ± standard error.

One-way ANOVA, followed by the Tukey’s honest significant difference post hoc test for pairwise comparisons, was used to detect changes in extrusion rate with different M250 concentrations and between the three incubation times. Statistical significance was set at *p* < 0.05.

The semi-lethal LC_50_ concentration of M250 was calculated using probit analysis [37].

## 3. Results

### 3.1. The Effect of M250 on N. bombycis Polar Tube Extrusion

Since tests for the viability of microsporidian spores based on the use of dyes such as trypan blue, propidium iodide, 4′,6-diamidino-2-phenylindole, etc., do not work without destroying the impermeable spore wall by special physical or chemical treatments [38], we determined this by measuring the percentage of polar tubes discharged after activation of this process [39,40]. According to our observations, the extrusion of *N. bombycis* polar tubes begins immediately after the spores are transferred from alkaline to neutral conditions and ends after approximately 1.5 min.

**Figure 2:**
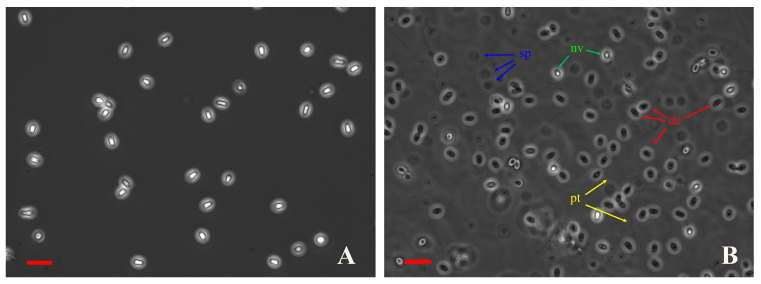
Discharged and undischarged *N. bombycis* spores look dark and light, respectively, in phase-contrast optics. (**A**) Intact spores without polar tube extrusion stimulation. (**B**) Control (untreated with antiseptics) spores after polar tube extrusion stimulation. ds—discharged spores, nv—non-viable spores, sp—sporoplasms, and pt—polar tubes. Scale bars 10 µm.

Control spores without antiseptic treatment showed an extrusion rate of almost 91% after their incubation in alkaline conditions followed by neutralization. On such slides, many discharged spores looked dark in the phase-contrast optics, surrounded by extruded polar tubes and sporoplasms, and only a few were light (Figure 2B). When polar tube extrusion was not stimulated, all spores looked light, like non-viable spores, and no polar tubes or sporoplasms were found in the medium (Figure 2A).

**Figure 3:**
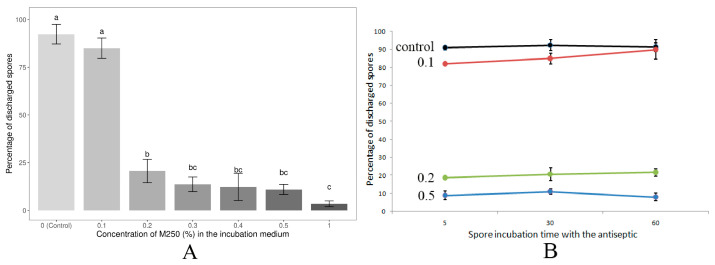
Dependence of *N. bombycis* spore viability (percentage of discharged spores) on M250 concentration (**A**) and incubation time (**B**). (**A**) Data from three independent experiments were analyzed. Bars represent the mean ± standard error. Different letters indicate statistically significant differences between groups analyzed by one-way ANOVA followed by Tukey’s post hoc test. The adjusted *p*-value for the difference between samples is less than 0.00001 for groups a and b, and a and c, and less than 0.01 for groups b and c. Double letters (bc) indicate that the values in these samples are not significantly different from b and c, but are different from a. (**B**) Control—spores without M250 treatment; 0.1, 0.2 and 0.5—the M250 content (%) in the incubation medium. Data are presented as the mean of three replicates ± standard error.

Treatment of *N. bombycis* spores with the experimental compound M250 at a concentration of 0.1% for 30 min showed no statistically significant differences in polar tube extrusion compared to control spores not treated with an antiseptic (Figure 3A). Increasing the M250 concentration to 0.2% resulted in an approximately four-fold loss of spore viability. A further increase in the M250 content in the incubation medium to 0.3–1.0% was also associated with a decrease in spore viability, but statistically significant differences were only observed when spores were treated with the antiseptic at concentrations of 0.2% and 1%. The semi-lethal LC_50_ concentration calculated by probit analysis corresponded to an M250 concentration of 1730 mg/L or 0.173%. Incubation times of 5, 30 and 60 min with M250 at concentrations of 0.1%, 0.2% or 0.5% did not show a statistically significant effect on the level of polar tube extrusion (Figure 3B).

### 3.2. Effect of M250 and Chlorhexidine Treatment of N. bombycis Spores on the Growth of Microorganisms Contaminating the Culture Media

As the 0.1% solution was the maximum concentration of M250 that did not affect polar tube extrusion, and therefore could be used to treat *N. bombycis* spores in cell cultures infected with microsporidia, we compared the efficacy of this compound with the most common commercial concentrations of the widely used biguanidine antiseptic chlorhexidine, which has a broad spectrum of antibacterial and antifungal activity [41] (Table 1).

As expected, the control spores, which were placed in the culture medium without any treatment, caused contamination in 100% of cases (Table 1). The addition of the antibiotic–antimycotic composition also failed to prevent the growth of undesirable microorganisms in the culture media of all of the wells tested. At the same time, bacterial development was observed in only 33% of the wells for spores previously treated with 0.1% M250 or 2% chlorhexidine. Spores treated with 0.05% chlorhexidine solution caused contamination in 67% of the wells tested. In all variants with spores treated with both antiseptics, the addition of antibiotics into the medium completely inhibited the development of unwanted microorganisms. A substantial disadvantage of chlorhexidine compared to M250 is that it completely blocks the polar tube extrusion of *N. bombycis* spores, even at 0.05% solution, which has also the worst disinfection effect.

## 4. Discussion

The main objectives of this study were (1) to evaluate the efficacy of the new antiseptic M250 in preventing the growth of fungi and bacteria contaminating Sf9 cell cultures infected with *N. bombycis* spores, and (2) to determine treatment conditions under which microsporidian spores, but not unwanted microorganisms, remain viable. Since the viability of microsporidian spores is directly related to their ability to transmit the sporoplasm through the extruded polar tube into a new host cell, spores that have lost this ability can hardly be considered as viable [42]. Therefore, this study used stimulation and visualization of polar tube extrusion to test the viability of microsporidian spores treated with the antiseptic M250.

This compound is a broad-spectrum disinfectant from the polymeric guanidine family with fast-acting antimicrobial activity. It is active against a number of medically important Gram-positive and Gram-negative bacteria at a minimum bactericidal concentration of about 1 mg/L, and against the pathogenic fungi *Candida* sp. and *Aspergillus* spp. at a minimum fungicidal concentration of about 0.5 mg/L [35,43]. The derivative of Mul-1867 is also a promising antifungal agent against phytopathogenic fungi such as *Fusarium oxysporum,* with a minimum fungicidal concentration of about 64 mg/L. After 30 min of treatment, it inhibited conidial germination and germ tube elongation of this phytopathogen [44].

The bactericidal and fungicidal activity of Mul-1867 was confirmed by transmission electron microscopy, suggesting that the death of these microorganisms is the result of membrane disruption [35,43]. Guanidine groups are known to bind to negatively charged molecules such as the carboxyl group (-COOH) of a fatty acid on the surface of bacteria, and hydrazine reacts with carbonyl groups. The binding of guanidine and hydrazine groups to phospholipids causes bacterial death and lysis [45]. The electron-dense proteinaceous exospore (the outer layer of the microsporidian spore wall) [46] may be more resistant to guanidine and hydrazine derivatives of Mul-1867 (the main component of M250) than the surface of bacteria or the cell wall of fungi.

Here, we demonstrated that M250 does not affect the ability of activated microsporidian *N. bombycis* spores to extrude their polar tubes at a concentration of 0.1%. Since this value is more than 1000 times higher than bactericidal and fungicidal concentrations of this compound, the effectiveness of spore treatment with M250 in preventing contaminating growth of microorganisms in microsporidia-infected cell cultures was expected and confirmed in this study. At the same time, it should be noted that antiseptic treatment of microsporidian *N. bombycis* spores with 0.1% M250 or 2% chlorhexidine alone is not sufficient to ensure the complete sterility of infected cultures. Apparently, they still contain bacterial spores that are resistant to these antiseptics. However, the further addition of antibiotics to the culture medium can completely block the development of contaminating microorganisms. At the same time, our study showed that the use of antibiotics alone is not able to suppress bacterial and fungal growth, although antibiotics are widely used in the establishment of cell cultures infected with microsporidia [47,48,49,50]. This is usually accompanied by the isolation of spores under aseptic conditions or thorough purification in density gradients [51].

An important advantage of the novel antiseptic over traditional chlorhexidine is the complete retaining of spore viability after treatment with M250 at an effective concentration of 0.1%. When spores were treated with chlorhexidine, their ability to extrude polar tubes was impaired, even at lower than effective concentrations. Thus, the novel antiseptic M250 at a concentration not exceeding 0.1% can be successfully applied for the treatment of microsporidian spores non-sterilely isolated from infected host animals to establish long-term cell cultures infected with these parasites. In our recent studies, we used 0.1% M250 to sterilize *Vairimorpha (Nosema) ceranae* and *N. bombycis* spores obtained from artificially infected insects, and these treatments did not affect the intracellular growth of either parasite [52,53,54].

In addition, treatment of *N. bombycis* spores with M250 at concentrations above 0.2% and especially above 1% showed a significant reduction in their ability to extrude polar tubes. As *N. bombycis* poses a great threat to sericulture, it is possible that the use of M250 at concentrations above 0.2% will be a promising replacement for the antiseptics traditionally used to disinfect silkworm nurseries, such as 10% formaldehyde and 10% oxalic acid.

## Figures and Tables

**Table 1:** Effect of antiseptics and antibiotics on culture medium contamination and spore viability. Each variant was analyzed independently three times in 6 different wells (n = 18).

Spore Treatment	Number of Wells with Contaminated Media out of 18 Analyzed Ones	Spore Discharge
Untreated	18 (100%)	+
Untreated + antibiotics	18 (100%)	+
0.1% M250	6 (33%)	+
0.1% M250 + antibiotics	0	+
2% chlorhexidine	6 (33%)	−
2% chlorhexidine + antibiotics	0	−
0.05% chlorhexidine	12 (67%)	−
0.05% chlorhexidine + antibiotics	0	−

## Data Availability

Data are contained within the article.
